# Draft Genome Sequences of Three Streptomycetes Isolated from Frobisher Bay Marine Sediments

**DOI:** 10.1128/mra.00604-22

**Published:** 2022-09-26

**Authors:** Anna I. Kuznetsova, Bradley A. Haltli, Russell G. Kerr

**Affiliations:** a Department of Biomedical Sciences, Atlantic Veterinary College, University of Prince Edward Island, Charlottetown, Prince Edward Island, Canada; b Department of Chemistry, University of Prince Edward Island, Charlottetown, Prince Edward Island, Canada; c Nautilus Biosciences Croda, Charlottetown, Prince Edward Island, Canada; Montana State University

## Abstract

Three *Streptomyces* strains (RKAG290, RKAG293, and RKAG337) were isolated from intertidal marine sediments of Frobisher Bay (Canada).

## ANNOUNCEMENT

Three *Streptomyces* strains were isolated from intertidal marine sediments collected from Frobisher Bay, Nunavut, Canada (63.72804°N 68.41989°W). The strains were isolated at 22°C using a heat-dried sediment stamp method and dilution plating on raffinose histidine agar (RKAG290) (1), chitin agar (RKAG293) (2), and *Streptomyces* isolation medium (RKAG337) (3) supplemented with 18 g/L Instant Ocean. The strains were identified as Streptomyces laculatispora (RKAG290, 1,523 bp with 99.93% identity to *S. laculatisopora* BBK166^T^ [accession no. FR692106.1]) and Streptomyces fildesensis (RKAG293, 1,519 bp, and RKAG337, 1,520 bp, with 99.58% identity to *S. fildesensis* GW25-5^T^ [accession no. DQ408297.1]) using full-length 16S rRNA gene sequences extracted from the genome sequence and the EZBioCloud 16S identification tool (v.2021.07.07) with default parameters (4).

Genomic DNA was obtained from lab cryostocks following the initial isolation with further culture reactivation in Difco ISP2 broth containing 1.8% Instant Ocean, incubated at 30°C, and shaken at 200 rpm for 4 days. Genomic DNA was prepared using the DNeasy UltraClean microbial DNA isolation kit (Qiagen). Library preparation and DNA sequencing were carried out by McGill University and Génome Québec Innovation Centre (Montréal, Canada) using a standard PacBio protocol for sheared large-insert (20-kb) libraries and a PacBio RS II single-molecule real-time platform (SMRT) with P4-C2 sequencing chemistries (Pacific Biosciences, USA) (5). Contig assembly was conducted using the Hierarchical Genome Assembly Process (HGAP v.1.4) (6) with a 30× cutoff. A subread length cutoff value was extracted from subreads and used in the preassembly step (BLASR v.5.3) (7), which consisted of aligning short subreads on long subreads. Long corrected reads were aligned and used as seeds in the assembly (Celera Assembler v.8.3rc2) (8) to generate contigs. These contigs were then “polished” by aligning raw reads on contigs (BLASR) that were then processed through the Quiver algorithm (6).

The characteristics of the draft assemblies of the genomes are summarized in Table 1. Annotation of the genomes using the NCBI Prokaryotic Genome Annotation Pipeline (PGAP v. 6.1) (9), yielded 7,055 to 8,107 predicted protein-coding sequences (CDSs), 69 to 73 tRNAs, and 18 rRNA genes per strain. Biosynthetic gene clusters (BGCs) encoding the biosynthesis of putative antimicrobial metabolites were identified with AntiSMASH v.6.0 (10) using default parameters, resulting in the identification of 23 (RKAG290), 32 (RKAG293), and 34 (RKAG337) BGCs. Cold shock proteins (CSPs) help cells cope with low temperatures and adapt to fluctuating environmental conditions (11). PGAP annotation identified 8 (RKAG290), 15 (RKAG293), and 8 (RKAG337) CSP-encoding genes. Future research will examine the role of CSPs and secondary metabolic BGCs in the temperature-dependent production of antimicrobial agents by these strains.

**TABLE 1:** Summary of genome assembly and annotation characteristics of three *Streptomyces* strains^*a*^

Parameter	Result for:		
	RKAG290	RKAG293	RKAG337
No. of reads	45,285	107,357	67,301
Avg subread length (bp)^*b*^	14,042	12,651	13,650
Length (bp)	8,105,749	9,268,343	8,055,297
GC content (%)	70.86	70.88	71.14
No. of contigs	26	3	4
Depth (×)	74	141	110
*N*_50_ (bp)	7,894,875	9,119,197	7,818,265
No. of:			
CDSs	7,096	8,107	7,055
tRNA genes	69	70	73
rRNA genes	18	18	18
CSPs	8	15	8
No. of BGCs			
Total	23	32	34
Terpene	5	4	4
Siderophore	1	2	2
NRPS	4	6	7
PKS			
Type 1	4	3	4
Type II	3	2	2
Type III	1	1	1
Lantipeptide	1	1	2
Other classes	4	10	10

a CSP, cold shock protein; BGCs, biosynthetic gene clusters; NRPS, nonribosomal peptide synthetase; PKS, polyketide synthase.

b Subreads that did not pass QC filtering were excluded from this calculation.

### Data availability.

This whole-genome sequencing project has been deposited in GenBank under accession no. PRJNA779104. Raw sequencing data sets were deposited for each strain separately for *S. laculatisopora* RKAG290 (SAMN22570722), *S. fildesensis* RKAG293 (SAMN22843389), and *S. fildesensis* RKAG337 (SAMN22843393). The raw sequences have been deposited in the NCBI SRA database under accession no. PRJNA779104.
